# Preclinical safety and efficacy of “Yubivaks,” a natural ointment for burn wounds: acute dermal toxicity and irritancy evaluation

**DOI:** 10.1590/1806-9282.20250666

**Published:** 2026-03-30

**Authors:** Susanna Babken Poghosyan, Karen Avetik Akopian, Siranush Hovannes Ter-Zaqaryan, Susanna Arshavir Muradyan, Anna Araik Keshishyan, Natalya Stepan Tadevosyan

**Affiliations:** 1 Yerevan State Medical University named after M. Heratsi, Research-Scientific Center, Laboratory of Environmental Hygiene and Toxicology – Yerevan, Armenia.; 2 Yerevan State Medical University named after M. Heratsi, Department of Maxillofacial Surgery – Yerevan, Armenia.; 3 Yerevan State Medical University named after M. Heratsi, Scientific-Educational Center for Fundamental Brain Research, Neuroscience Laboratory – Yerevan, Armenia.

**Keywords:** Safety, Burn, Healing, Sea buckthorn oil, Propolis, Beeswax, Clove oil

## Abstract

**OBJECTIVE:**

Natural medicines are considered an effective strategy for treating burns. A preclinical study of a new natural ointment, “Yubivaks,” was conducted to study acute dermal toxicity and irritant properties to ensure its safety.

**METHODS:**

The study investigated the acute dermal toxicity, local irritant effect on the skin and mucous membranes of eyes, and the sensitizing activity of the ointment. Adult nonlinear white rats and rabbits of both sexes were used in the experiment. The rats were randomly divided into three groups, each consisting of 10 animals: group I received recommended therapeutic dose (0.003 kg/day), group II received a “conditionally toxic” dose (0.020 kg /day), and group III served as the control group.

**RESULTS:**

There were no deaths or signs of intoxication in the acute dermal toxicity study. No local irritant, resorptive, or sensitizing effect and positive immunological tests were observed at the therapeutic dose. Treatment of experimental burns with “Yubivaks” reduced the wound surface by half on the 7th day (59.6%); on the 15th day, the restoration of the histostructure of the epidermal tissue was 99.8%; and by the 25th day, the burn area was completely restored (100%) compared to “Levomekol”: 43.6, 51.1, and 95.8%, respectively. Comparison with “Levomekol” was made based on its wide use as a recognized traditional medicine with complex action.

**CONCLUSION:**

At the recommended therapeutic dose, the ointment can be considered as an alternative medication due to accelerated regenerative processes, pronounced anti-inflammatory properties, and safety proven in experimental studies. A limitation of the study was that it was only conducted on rats and rabbits, without using guinea pigs or pig models for several reasons.

## INTRODUCTION

Burn injuries of both chemical and thermal origin are a significant cause of morbidity and mortality worldwide. In the past, they were initially treated with natural remedies such as plants, oil, honey, and the like^1,2^. Although a global decline in burn incidence has been observed, mortality rates remain high. According to the World Health Organization, burns cause about 180,000 deaths per year. Globally, there has been a decline in burn incidence, severity, burn mortality, and length of hospital stay, especially in developed countries^3,4^.

In clinical practice, various agents are used to treat burn wounds, which are effective and have a wide range of action^1^. Along with this, researchers and practitioners have increasingly begun to pay attention to products based on natural substances, which are a source of biologically active substances and, as a rule, have a fairly high efficiency, a wide range of therapeutic effects, and low toxicity. Other advantages of herbal remedies include low cost, availability, and fewer side effects. Many studies are being conducted around the world to identify and isolate the active components of medicinal plants that have wound-healing properties. Medicinal plants can act as wound-healing agents due to a wide range of their various components such as alkaloids, essential oils, flavonoids, tannins, terpenoids, phenolic compounds, and so forth, which have the potential to improve the healing process of burn wounds. The wound-healing process consists of re-epithelialization, granulation, and neovascularization, which lead to wound contraction. Phytochemicals can influence different stages of this process through different mechanisms.

In this regard, a review of the research findings by Bahramsoltani et al. aimed to evaluate various single- and multi-component herbal preparations and their phytochemical components for burn wound-healing properties. Among the single herbal preparations, *Allium sativum*, *Aloe vera*, *Centella asiatica*, and *Hippophae rhamnoides* were shown to exhibit the best evidence for their wound-healing effect through different mechanisms. Among the combined herbal preparations, the most effective were Ampukar, a topical oil-based preparation, and a combination of five Iranian medicinal plants that stimulate wound healing. The wound-healing process consists of re-epithelialization, granulation, and neovascularization, which lead to wound contraction. Phytochemicals can influence different stages of this process through different mechanisms^5^.

While the ancient Egyptians and Greeks used honey to treat wounds, around the world, a wide range of wounds are treated with natural, raw honey from a variety of sources; “MediHoney” is one of the first medically certified honeys licensed as a medical product for professional wound care. “MediHoney” is a Manuka honey-based product that consists of a standard mixture of two honeys derived from Australia and New Zealand, containing glucose oxidase and different *Leptospermum* spp. honeys. It is an FDA-approved natural remedy offered and used for the treatment of various wounds including diabetic foot ulcers, pressure ulcers, burns, and the like, which exhibits standard antibacterial activity as confirmed by appropriate in vitro testing methods. Compared to “Yubivaks,” which is a mixture of a number of natural active ingredients, “MediHoney” is basically a single herbal preparation consisting primarily of 80% active *Leptospermum honey* (also known as Manuka honey) and 20% natural gelling agents, and may also include natural plant waxes^6^.

There is no doubt that interest in herbal medicine has grown and will undoubtedly continue to grow worldwide. Herbal medicine is expanding, and the public and healthcare consumers worldwide continue to incorporate it into health choices^7^. In clinical trials, it was shown that some herbal preparations have better effectiveness in treating burn wounds, including shortening the healing time and reducing inflammation, than the conventional treatment used hitherto. Research on animal models shows that many extracts may potentially benefit the treatment of burn wounds and sunburn. Due to the diverse mechanism of action, antibacterial activity, safety of use, and cost-effectiveness, herbal preparations can compete with conventional treatment^8^.

The integration of natural products into burn treatment protocols not only enhances the healing process but also supports the overall well-being of patients by reducing the likelihood of adverse reactions and promoting a more natural recovery process. The WHO has reported that 70–95% of the population in developing countries relies on medicinal plants for primary healthcare. Over recent decades, there has been a growing scientific interest in the application of medicinal plants in wound treatment, highlighting their potential in modern therapeutic practices. It has been estimated that a significant proportion of wound-healing agents in traditional medicine, particularly in Ayurvedic practices, are derived from plant sources, with approximately 70% of these remedies being plant-based. Mineral-based remedies constitute around 20%, and the remaining 10% are derived from animal products^9^.

In this regard, the proposed “Yubivaks” is new, compared to existing natural ointments, as it is a mixture of a number of natural active ingredients: *H. rhamnoides*, *propolis*, *beeswax*, and *clove oil*, present in a specific ratio. These ingredients exhibit identical, unidirectional biological activity, which is enhanced when they are combined. The proposed component ratio promotes accelerated regenerative and anti-inflammatory processes, which have been proven by experimental studies on animal models. The aim of this study was to investigate the acute skin toxicity and irritant properties of “Yubivaks” ointment to ensure its safety in burn treatment.

## METHODS

### Materials

**Figure 1.**
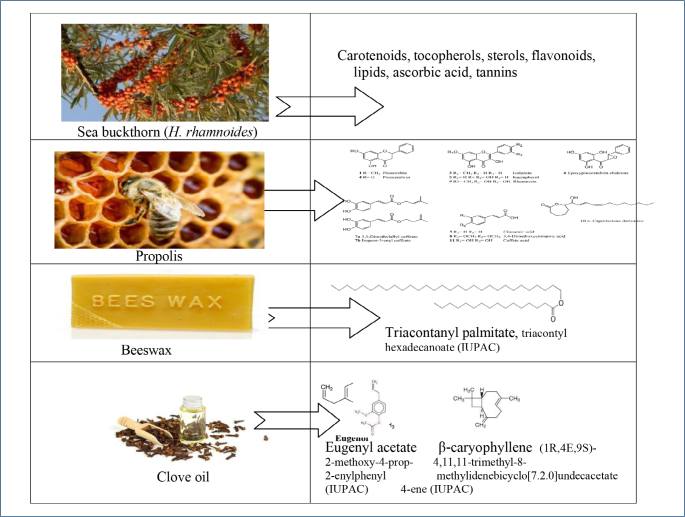
Active ingredients of “Yubivaks.”

The ointment “Yubivaks” was developed at the Department of Maxillofacial Surgery of the Yerevan State Medical University named after M. Heratsi (Armenia) and is proposed for the treatment of chemical and thermal burns of II to III degrees (T20-T32, IDC-10)10. The ointment “Yubivaks” contains natural components: *sea buckthorn oil* or *H. rhamnoides* (CAS 225234-03-7 / 90106-68-6), *propolis* (CAS 9009-62-5), *beeswax* (CAS 8012-89-3), and *clove oil* (CAS 8000-34-8), which have multifaceted biological activity (Figure 1).

The active components of “Yubivaks” are in the following proportions: sea buckthorn oil (86–88%), beeswax—(7–8%), propolis (1.5–1.9%), and clove oil (1.75–2.1%)^10^. The medicinal properties of natural components are due to the presence of antioxidants, vitamins, carotenoids, tocopherols, fatty acids, and other biologically active substances^11,12,13^. They have a wide range of antibacterial, antiviral, antifungal, antitumor, anti-inflammatory, and antioxidant effects^14,15^.

### Animals and toxicity study

The study was approved by the Ethics Committee of the Yerevan State Medical University after M. Heratsi (No. 12, 16.06.2016). The procedures conform to the EU-adopted Directive 2010/63/EU of the European Parliament and of the Council on the protection of animals used for experimental and other scientific purposes, observing all requirements for the maintenance, care, and feeding of laboratory animals^16^. Studies were done on adult nonlinear white rats and rabbits of both sexes. Prior to the study, the animals were quarantined and clinically healthy. Male and female rats were housed separately in the vivarium of Yerevan State Medical University under standard conditions. Animals were monitored daily for mortality and changes in fur, skin, eyes, gait, and posture.

For the acute dermal toxicity test, rats were randomly divided into three groups, each contaning 10 animals of both sexes: group I received the recommended therapeutic dose of 0.003 kg/day, group II was administered a “conditionally toxic” does of 0.020 kg/day, and group III served as the control group. The choice of 0.003 kg/day for the “Yubivaks” ointment as the recommended therapeutic dose was based on preclinical studies conducted on an experimental thermal burn model. The “conditionally toxic” dose of 0.02 kg/day was determined based on the maximum amount of “Yubivaks” needed to create a suspension in saline. The sample size (n=10/group) adhered to guidelines from several sources, and the study was conducted in accordance with their requirements^17,18,19^. Animals were housed individually in plastic cages. Notably, two-thirds of the tail (~5% of the body surface area) of each rat was immersed once in an emulsion of ointment at the appropriate dose, while rats in the control group were exposed to saline solution. The duration of exposure was 4 h. Visual observations were made to assess mortality and behavioral patterns such as food and water consumption, as well as reactions to tactile and pain stimuli. These observations were conducted at the end of the exposure and then daily for 14 days. Body weight, erythrocytes and leukocyte counts, and hemoglobin levels were measured on the 1st and 14th days. Additionally, macroscopic examination of the internal organs of the animals was performed at the end of the experiment (14th day). According to approved guidelines, the duration of observation for animals in acute toxicity studies should be at least 14 days, with continuous monitoring on the first day following administration^18^.

The local irritant effect was also studied in rats randomly divided into three groups with 10 animals of both sexes in each group. The ointment was applied in an even layer to the clipped side of the rats (5% of the total body surface) using an ophthalmic glass spatula. The exposure time was 4 h, and applications were carried out daily for 4 weeks (20-fold applications). The doses corresponded to those used to assess acute dermal toxicity. Control group III was similarly tested with saline^18^. The possible primary skin reaction was assessed immediately after application, after 15 and 30 min, and then after 1, 3, 24, 48, 72 h, and until the end of the experiment. The skin status, such as redness, swelling, cracks, hemorrhages, and the formation of a dry crust, as well as signs of hyperemia, erythema, and edema, was monitored. The edema was determined by measuring skin fold thickness.

An assessment of the ointment’s irritant effect on the eyes’ mucous membranes was carried out on rabbits (n=6). A single application of ointment in a dose of 50 mg was applied to the conjunctival sac of the eye, and the opposite eye served as a control. After 1 h, daily observation was conducted to monitor the cornea and the mucous membrane status for 14 days.

The possible allergenic effect of the ointment was studied for 30 days on rats divided into three groups (n=10) in accordance with the approved complex sensitization scheme, and skin reaction was assessed using the Magnusson and Kligman scale^19^.

### Efficacy assessment

In preclinical studies on the model of experimental thermal burn, a histopathological assessment of the effectiveness of topical application of “Yubivaks” ointment, as well as the dynamics of changes in the microvascular system of burn wounds, was carried out, and re-epithelization, wound closure rates, and restoration of the histostructure of the epidermal tissue were studied^20,21,22^.

### Statistical analysis

Statistical analyses were performed using the standard software (Microsoft Excel). Quantitative data were expressed as the mean±standard error. Standard errors and other indicators were calculated using the Litchfield-Wilcoxon probity analysis method in Prozorovkey modification^23^. Results were compared between the treatment and control groups and were analyzed using a two-tailed Student’s t-test for independent samples (t-test) with Bonferroni correction; significance was considered at a p-value less than 0.017. The hazard class was established in accordance with the criteria for assessing the degree of impact on the human body^24^.

## RESULTS

It was not possible to establish a dermal LD_50_ value. Most likely, it exceeds the level of the “conditionally toxic” dose: 0.020 kg/day. The acute skin toxicity assessment indicated that no death of animals or visible signs of intoxication were observed either in group I (0.003 kg/day) or in group II (0.02 kg/day). The general condition of the animals under study was consistently satisfactory. No changes in behavioral reactions or the condition of the skin of the tail and body were noted. There were no significant changes in some studied parameters, p>0.05 (Table 1). Macroscopic examination of the internal organs of animals in the experimental group did not reveal any changes. The absence of any changes in the internal organs during macroscopic examination served as the basis for not conducting a histopathological examination of the internal organs (e.g., liver, kidneys).

**Table 1:** Some variables of acute dermal toxicity of “Yubivaks” ointment in the treatment and control groups of experimental animals.

Variables and terms^a^	Treatment group I: therapeutic dose, 0.003 kg/day	Treatment group II: “conditionally toxic,” 0.020 kg/day	Control group III
Body weight, kg			
in 24 h	0.238±0.0007	0.238±0.0009	0.238±0.0009
in 14 days	0.240±0.0004	0.239±0.0005	0.240±0.0007
	p>0.05^*^	p>0.05^*^	
Red cells, 10^12^/L			
in 24 h	4.91±0.09	4.95±0.07	5.03±0.11
in 14 days	4.98±0.07	4.93±0.04	5.01±0.09
	p>0.05^*^	p>0.05^*^	
Leukocytes, 10^9^/L			
in 24 h	10.8±0.4	10.12±0.5	10.15±0.5
in 14 days	10.6±0.7	10.28±0.9	10.17±0.5
	p>0.05^*^	p>0.05^*^	
Hemoglobin, G/L			
in 24 h	129.0±1.00	131.0±0.35	131.7±0.89
in 14 days	129.2±0.80	131.2±0.37	131.7±0.97
	p>0.05^*^	p>0.05^*^	

^a^The data is presented as mean±standard error (SE).

*p-values obtained in comparing treatment and control groups.

In studying the local irritant effect after repeated applications of the ointment, no manifestations of skin damage in the form of hyperemia, erythema, cracks, ulcers, or hemorrhages were detected (p>0.05). A study of the irritant effect on the mucous membranes of the eyes of rabbits with a single application of the ointment to the conjunctival sac did not reveal any characteristic of serious irritations. A slight increase in the vascular pattern of the eyeball, slight reddening of the conjunctiva, hyperemia, and lacrimation were observed, and within 72 h of observation, the test animals fully recovered. Based on the results of this test, the ointment is classified as a mildly irritating substance.

The results obtained in complex sensitization testing made it possible to evaluate skin reactions in rats of group I at 0 points (no visible changes) on the Magnusson and Kligman scale.

The initial comprehensive assessment of the sensitizing effect of the ointment (in vitro) did not reveal any allergenic effect at the therapeutic dose.

## DISCUSSION

Burn wound healing is a complicated process including inflammation, re-epithelialization, granulation, neovascularization, and wound contraction. Although several preparations are available for the management of burn wounds, there is still a necessity for researching efficacious medicine^5,25^. In recent decades, there has been increased interest among researchers and medical professionals in the study and use of herbal remedies— phytotherapeutic agents. This growing interest in natural-based preparations is driven by their ability to provide biologically active substances without associated toxicity or side effects. This interest may also be driven by the potential risks of irrational use of allopathic medications, as well as their high cost. The World Health Organization recognizes that approximately 85% of the population in developing countries uses plants or herbal products for medicinal purposes^26,27^.

The ointment “Yubivaks” was subjected to preclinical testing on a model of experimental chemical and thermal burns. The ointment consisting of natural components in the proposed ratio, such as sea buckthorn, clove oils, beeswax, and propolis, has multifaceted biological activity. The ointment is new compared to existing natural ointments, since it is a mixture of a number of active ingredients with the same unidirectional biological activity, which, in total, apparently, is enhanced. The enhanced healing properties were registered in the treatment of experimental burns with “Yubivaks.”

A histological assessment of the effectiveness of “Yubivaks” ointment, as well as the dynamics of changes in the microvascular system of experimental burn wounds, was carried out, such as re-epithelization, wound closure rates, and restoration of the histostructure of the epidermal tissue. In preclinical studies of the ointment, “Yubivaks” at the recommended therapeutic dose on the model of experimental burn, already on the 7th day, a significant decrease in the number of neutrophils and an increase in the number of fibroblasts, lymphocytes, and macrophages in the wound were observed; rapid and uniform formation of granulation tissue throughout the lesion and a high density of newly formed vessels were noted, which contributed to the activation of regeneration processes compared to the use of “Furacilin” and “Levomekol” ointments. Comparison with these ointments was made based on their wide use as a recognized traditional medicine with complex action. The obtained results have shown that the wound surface was reduced by half on the 7th day, amounting to 6.2%, on the 15th day, with complete restoration of the histostructure of the epidermal tissue on the 21st day, compared with “Levomekol”: 58.3, 17.89, and 4.54%, respectively. In the experimental burn model, the widely used ointments “Furacilin” and “Levomekol” were used as positive controls for “Yubivaks,” by the 25th day of observation, the burn area had completely recovered with “Yubivaks” (100%), compared to 98 and 95.8% with “Furacilin” and “Levomekol,” respectively. On the 25th day, in experimental animals treated with “Yubivaks,” the histological structure of the burn injury resembled the structure of healthy skin, and islands of granular tissue in the depths of the lesion were practically not detected. After 90 days, the burn area was overgrown with thick fur^20,21,22^. Research results have shown that, due to the identical healing properties of its components, “Yubivaks” ointment has a powerful regenerative effect on burn wounds, promoting intensive and rapid healing compared to traditionally used ointments such as “Furacilin” and “Levomekol.”

An acute dermal toxicity study revealed no deaths, signs of intoxication, or any skin changes at the recommended therapeutic dose (0.003 kg/day). The absence of any changes in the internal organs during macroscopic examination of the internal organs of animals in the experimental groups served as the basis for not conducting a histopathological examination of the internal organs (e.g., liver, kidneys) and the claim of “no toxicity” of “Yubivaks” ointment. In these terms, “Yubivaks” is classified as a class 5 hazard substance. The weak irritant effect on the mucous membranes of the eyes allowed the ointment to be classified as hazard subclass 2B (mildly or slightly irritating). The assessment of primary sensitization in vivo and negative results of immunological reactions in vitro allowed it to be classified as subclass 1B (low potential for sensitizing action upon contact with skin).

The medicinal properties of “Yubivaks” are explained by the well-known medicinal activity of its ingredients. In this regard, our study is in line with a number of studies dedicated to medicinal plants. Several studies have reported the effectiveness of a number of herbal remedies such as olive oil, clove oil, and beeswax, as well as propolis and its various mixtures, in the treatment of wounds. Many Asian systems use local herbal sources as medicines for treating chronic wounds, which are often more readily available. When considering the issue of access to ancient remedies, it is worth noting that they are available locally, whereas most of the new Western knowledge and new remedies are not available to most countries in the developing world^28^.

There are different approaches to herbal remedies, and they must be respected and valued. In research assessing the opinion of health professionals on the use of phytotherapics in public health care of Basic Health Units, 60% reported having information only from popular culture and only 20% obtained their knowledge from journals. The survey data from physicians clearly demonstrated the lack of awareness of professionals about numerous studies published in national or international journals, as well as the insufficient knowledge that could be obtained within their academic programs. A study examining the inclusion of phytotherapy in the curricula of Brazilian public universities noted interest among students and professionals in its use and study. Phytotherapeutic medicines, like any other medication, can have adverse and toxic effects. The authors believe that phytotherapics can be very useful as a comprehensive or complementary therapy for many clinical conditions, but further data on its safety and efficacy are needed to ensure confidence in such a powerful tool. Phytotherapeutic medicines should be viewed as an accessible medicine and a complementary treatment method, and not as a placebo^27^.

Because burns carry the risk of disability and death, much research is focused on their treatment, which remains complex and expensive. A clinical study was conducted by Abdullahzadeh et al. to evaluate the wound-healing period with sea buckthorn dressings for second-degree burns and compare the results with 1% silver sulfadiazine (SSD) dressings. It was concluded that the healing period in the group treated with sea buckthorn cream was shorter than in the group treated with 1% SSD (p<0.001). The results demonstrated the greater clinical efficacy of sea buckthorn cream over 1% SSD for healing second-degree burns. Using these natural products to treat burns can shorten the healing period, thereby potentially shortening the course of treatment and reducing the burden on health care services^29^.

Simon et al. examined “MediHoney” as an alternative wound treatment. This medicinal honey consists of a standard mixture of two honeys derived from Australia and New Zealand, containing glucose oxidase and different types of *Leptospermum* spp. honeys. It exhibits a standard antibacterial activity, confirmed by appropriate in vitro methods. Unlike “MediHoney,” “Yubivaks” is a mixture of several natural active ingredients that provide a comprehensive effect, promoting intensive and rapid healing of burn wounds^6^.

A study by Ismail et al. comparing the healing time of burn wounds using SSD, *A. vera*, *honey*, and amniotic membrane found that *A. vera* was the most effective (MD: -4.75; 95%CI -8.67 to -0.86), followed by amniotic membrane (MD: -4.71; 95%CI -7.45 to -1.97) and honey (MD: -4.25; 95%CI -6.76 to -1.73). The results showed that *A. vera* and amniotic membrane were effective in wound healing, and honey was particularly effective in preventing wound infection in burn patients^30^.

Seda Askin and Merve Kaynarpinar investigated the therapeutic effects of a spray containing a mixture of plant extracts from *A. vera*, *Olea europaea*, *Chamomilla recutita*, and *Cocos nucifera* (AOCC) in a second-degree burn model. The authors demonstrated the therapeutic effect of AOCC in terms of oxidative stress markers and gene expression levels regulating oxidative stress and inflammatory processes during wound healing. The AOCC spray mixture exerted a positive effect on burn-injured rats due to its constituent ingredients. These positive effects of AOCC can be attributed to its antioxidant, antiseptic, and anti-inflammatory properties^31^.

A study by Lopes et al. found that essential fatty acids are also effective in treating a range of conditions, including dermatological, renal, and visual problems, as well as fertility disorders. Essential fatty acids are polyunsaturated fatty acids (PUFAs) that are not synthesized in the body and are precursors to important substances involved in various functions. The results indicate that PUFAs are involved in a number of metabolic processes, providing not only preventative but also therapeutic benefits in a number of diseases. Studies have shown that in cases of dermatological, renal, visual, and fertility disorders that developed as a result of PUFA deficiency, the administration of these acids led to the elimination of symptoms^32^.

A review of articles on the use of traditional and complementary medicine, focusing on how they affect healing, found that the most common plants were *A. vera*, *C. asiatica*, *Arnebia euchroma*, and *H. rhamnoides* (sea buckthorn) seed oil, and that traditional medicine was useful in treating burn wounds. In other words, herbal preparations can be considered as an alternative in the treatment of burn wounds, as they affect the inflammatory response and reduce exudation, leading to an acceleration of the healing process^33,34^.

Thus, according to the results of preclinical studies of “Yubivaks” ointment, all ingredients of the proposed composition, due to their identical healing properties, exhibit a synergic effect, manifested in the activation of the re-epithelization process and anti-inflammatory action, resulting in a powerful regeneration effect on burn wounds, promoting their intensive and rapid healing. The findings obtained in the present study are fully consistent with the results of the above-mentioned studies aimed at evaluating the therapeutic effects of various herbal remedies on burn injuries.

This study has limitations. Studies of the sensitizing properties of “Yubivaks” were conducted only on adult nonlinear white rats, without the use of guinea pigs, for several reasons. One reason was the statement made in Test Guideline No. 406: Skin Sensitization: “Both the Buehler and Guinea pig maximization tests use animals. For animal welfare reasons, these tests should only be conducted as a last resort, if justified e.g., when other skin sanitization test methods are not applicable.” Therefore, for a more detailed study of sanitization properties, tests might also be conducted on guinea pigs.

## CONCLUSION

Based on the results obtained in the study of acute skin toxicity, local irritant effect on the skin and mucous membranes of the eyes, and sensitizing activity, as well as the results of the study of the healing process in an experimental burn model, it can be concluded that “Yubivaks” ointment is a safe and effective medicine. It can be used as an alternative medicine in the treatment of burn wounds due to its effectiveness because of the activation of re-epithelialization, acceleration of regenerative processes and wound healing, pronounced anti-inflammatory properties, and safety. At the recommended therapeutic dose, there is no risk of general toxic side effects.

## Data Availability

The datasets generated and/or analyzed during the current study are available from the corresponding author upon reasonable request.
